# Lichen sclerosus: The 2023 update

**DOI:** 10.3389/fmed.2023.1106318

**Published:** 2023-02-16

**Authors:** David A. De Luca, Cristian Papara, Artem Vorobyev, Hernán Staiger, Katja Bieber, Diamant Thaçi, Ralf J. Ludwig

**Affiliations:** ^1^Lübeck Institute of Experimental Dermatology, University of Lübeck, Lübeck, Germany; ^2^Department of Dermatology, University Medical Center Schleswig-Holstein, Lübeck, Germany; ^3^Department of Dermatology, Hospital Italiano de Buenos Aires, Buenos Aires, Argentina; ^4^Institute and Comprehensive Center Inflammation Medicine, University of Lübeck, Lübeck, Germany

**Keywords:** lichen sclerosus, kraurosis vulvae, white spot disease, balanitis xerotica obliterans, autoimmunity

## Abstract

Lichen sclerosus (LS) is an underdiagnosed inflammatory mucocutaneous condition affecting the anogenital areas. Postmenopausal women are predominantly affected and, to a lesser extent, men, prepubertal children, and adolescents. The etiology of LS is still unknown. Hormonal status, frequent trauma and autoimmune diseases are well-known associations for LS, yet infections do not seem to be clear risk factors. LS pathogenesis involves factors such as a genetic predisposition and an immune-mediated Th1-specific IFNγ-induced phenotype. Furthermore, there is a distinct expression of tissue remodeling associated genes as well as microRNAs. Oxidative stress with lipid and DNA peroxidation provides an enabling microenvironment to autoimmunity and carcinogenesis. Circulating IgG autoantibodies against the extracellular matrix protein 1 and hemidesmosome may contribute to the progression of LS or simply represent an epiphenomenon. The typical clinical picture includes chronic whitish atrophic patches along with itching and soreness in the vulvar, perianal and penile regions. In addition to genital scarring, and sexual and urinary dysfunction, LS may also lead to squamous cell carcinoma. Disseminated extragenital LS and oral LS are also reported. The diagnosis is usually clinical; however, a skin biopsy should be performed in case of an unclear clinical picture, treatment failure or suspicion of a neoplasm. The gold-standard therapy is the long-term application of ultrapotent or potent topical corticosteroids and, alternatively, topical calcineurin inhibitors such as pimecrolimus or tacrolimus. Collectively, LS is a common dermatological disease with a so far incompletely understood pathogenesis and only limited treatment options. To foster translational research in LS, we provide here an update on its clinical features, pathogenesis, diagnosis and (emerging) treatment options.

## 1. Introduction

Lichen sclerosus (LS) is a chronic mucocutaneous immune-mediated disease which typically involves genital skin. The term was first coined by Hallopeau in 1887 and it received multiple names such as kraurosis vulvae, balanitis xerotica obliterans, white spot disease, leukoplakia and lichen sclerosus et atrophicus. The final term “lichen sclerosus” was accepted in 1976 by the International Society of the Study of Vulvovaginal Disease (1, 2).

The etiology and pathogenesis of LS are still not fully elucidated. There is a genetic and familial predisposition in LS, whereas frequent trauma, hormonal status and certain drugs could also play a role in the pathogenesis. LS is a type 1 T helper (Th1) mediated and miR-155 dependent immune-mediated disease. Although autoantibodies against extracellular matrix protein 1 and BP180 have been described, it is still unclear if they represent an accurate piece of LS pathogenesis. There is also a particular expression of tissue remodeling associated genes and an exacerbated oxidative stress that may lead to scarring and malignancy (3).

A bimodal peak incidence of LS in premenarchal girls and in menopausal women has been outlined. However, premenopausal women could experience LS with mild symptoms, delaying the diagnosis for several years and underestimating the real incidence in this age group. Men and adolescents are also, to a lesser extent, affected by the disease (4, 5).

The clinical picture of anogenital LS (gLS) includes ivory-white patches, atrophy and severe pruritus, altering the quality of life (QoL). Extragenital LS (eLS) comprises areas such as neck, shoulders, upper trunk, thighs and oral cavity (6, 7). The lesions could evolve to scarring of the vaginal introitus, phimosis and functional impairment (8). Furthermore, it has been described as an intrinsic risk factor for malignancy in untreated patients, but there is no evidence of increased neoplasm development after long-term treatments (9, 10).

The established diagnosis of LS based on the clinical features is usually sufficient, and a skin biopsy should only be performed in case of clinical doubts, differential diagnosis or suspected malignancy. The first-line therapy is ultrapotent or potent topical glucocorticoids (TC) and in case of anatomical changes due to scarring, surgical procedures should be performed (3, 11). In order to prevent complications, aside from an early diagnosis and a correct treatment, a long-term follow-up is imperative (9). The objective of this review is to deliver a thorough update of LS focusing on its pathogenesis, clinical features, diagnosis and treatment options.

## 2. Epidemiology

LS can occur at any age and it affects both sexes. However, women are most commonly affected, with a female-to-male ratio between 3:1 and 10:1 (9). A family history of LS has been described in 8.7% of women affected by this condition (12). There is a well-known historically bimodal presentation of vulvar LS (VLS) with a first peak in prepubertal girls (average: 7.6 years) and a second one during the peri- and postmenopause (average age: 52.6 years) (13). This specific distribution in females has been linked to a low estrogen status, leading to a humoral over T-cell mediated response, as well as to a Köbner phenomenon due to the lack of proper lubrication (9). However, VLS can affect women of any age and the proportion in fertile women could be higher than expected. In a survey of women of reproductive age with histological confirmation of VLS, the mean age of diagnosis was 32 years, with symptom onset at 27 years (14). Furthermore, up to 40% of the surveyees described symptoms prior to menopause or they were diagnosed with asymptomatic VLS during the gynecologic examination (11, 15). On the contrary, the onset of male genital LS (MGLS) in men is relatively stable with a first postpubertal peak during the third decade and a second one after 60 years (16). The mean age of onset in adult men was 36.2 years and a delay to seek medical advice of 1.6 years (17).

The exact prevalence and incidence of LS are underestimated. On the one hand, one third of the cases are asymptomatic, while, on the other hand, LS is frequently misdiagnosed or unrecognized (4). The estimated incidence of LS in both sexes is 0.1 to 0.3% (8). In regard to VLS, the prevalence in general gynecology private practice reached 1.7% (15). The Research Institute of the Health Insurance AOK in Germany exhibited a prevalence of 0.29% in women over 80 years old, whereas the prevalence in nursing home women over 80 years old escalated to 3%, probably influenced by incontinence and immobilization (18, 19). According to the Brooke Army Medical Center, the incidence of LS in men reached 0.07%, while the Department of Defense in the USA reported an incidence of 0.0014% (16, 20).

The incidence of pediatric LS is estimated at 0.04 to 0.06%, with a female-to-male ratio of 1:1.7 (21, 22). In a systematic review, the age-onset in children was 6.5 years in females and 8.6 years in males, with a mean diagnostic delay of 18 and 12.5 months, respectively (22). Premenarchal VLS presents with nonspecific symptoms and can occur in 1:900 girls. These symptoms usually withdraw after the menarche, underestimating further the frequency of LS (23). In a prospective observational study, histologically diagnosed preputial LS was found in 32% of male children with phimosis. Boys with phimosis due to LS were older than those without the disease (mean 8.4 versus 4.7 years old) (24). In Germany, LS in prepubertal boys was estimated to have a much higher prevalence of up to 0.4% of, as the majority are not commonly circumcised after birth (25).

Other forms of LS are rare. It is estimated that eLS comprises only 15–20% of LS patients. It usually occurs simultaneously with gLS, but in 6% of the cases, eLS is present as an isolated entity without any genital lesions. Most cases of eLS are diagnosed in middle-aged adults (26, 27). Nevertheless, the prevalence of eLS may be underestimated,as it is also frequently asymptomatic (28).

## 3. Pathogenesis

**Figure 1:**
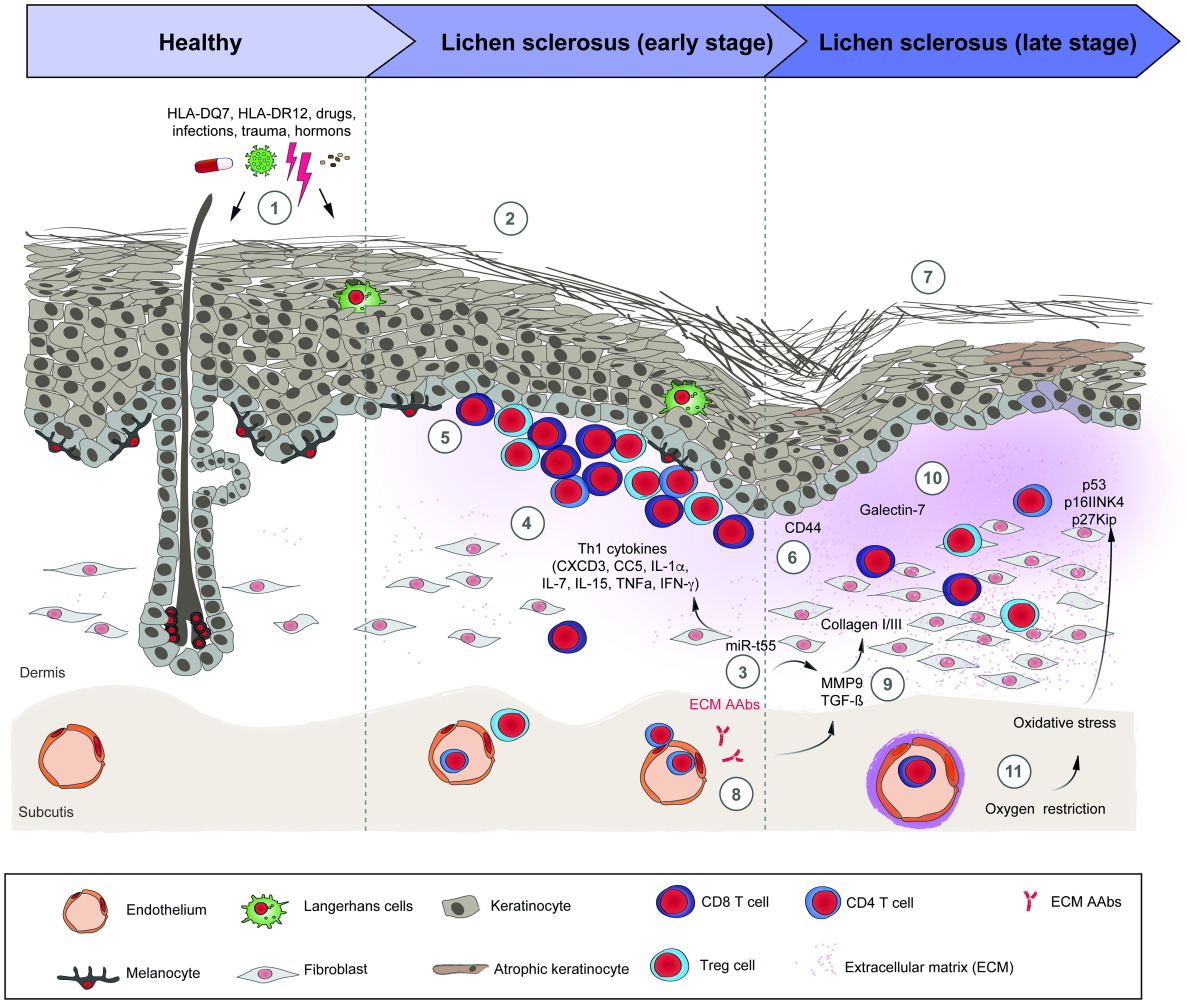
Schematic overview of lichen sclerosus pathogenesis. (1) Risk factors for LS; (2) The inflammatory early stage of LS is unspecific and shows a dermoepidermal interface band of mostly T-cells. (3) miR-155 is overexpressed in LS, stimulates the Th1 profile and the dermal sclerosis. (4) Th1 cytokines implicated in the pathogenesis of LS. (5) Lichenoid infiltrate of TCD4+, TCD8+, and Tregs cells in the upper dermis. (6) Abnormal expression of CD44 in the epidermis and lichenoid infiltrate. (7) The sclerotic late stage of LS presents an atrophic epidermis, with comedo-like plugs and a cleft in the stratum corneum, dermal sclerosis, reduction and dilation of dermal vessels and the appendages disappear. (8) The autoantibodies against EMC1 may activate MMP9, which subsequently activates TGF-β. (9) TGF-β and BMP2 induce the synthesis of collagen I and III in fibroblasts. (10) Galectin-7 induces the synthesis of collagen I and III. (11) Along with inflammation, the vessel sclerosis contributes to the oxidative stress in LS, leading to downregulation of tumor suppressors genes and overexpression of p53 in the skin, factors related with the development of skin carcinomas. The precise sequence of events and their interactions are only incompletely understood. References: BMP2: bone morphogenetic protein 2; EMC1: extracellular matrix protein 1; LS: lichen sclerosus; MMP9: metalloproteinase 9; TGF-β: tumor growth factor beta. TNF-α: Tumor necrosis factor alpha.

A summary of the pathogenesis and histological changes in LS is depicted in Figure 1.

### 3.1. Genetics

Immune-mediated diseases have an immunogenetic scenario. A positive family history of LS in first-degree female relatives can be found in 12% of patients (29). HLA-DR and DQ are supposed to be involved in the susceptibility and protection from LS (30). In children with VLS, HLA-DQ7 was present in 66% of the cases, in comparison to 31% in controls. Although these children showed a low association with autoimmunity, up to 56% of their relatives presented another autoimmune disease (31). In both adult male and female patients, HLA-DQ7 has been observed to occur more frequently in LS (32, 33). A study of UK women with VLS demonstrated an increased frequency of HLA-DR12 (DRB1*12) and a lower frequency of HLA-DR17 (DRB1*0301/04) compared to controls. Furthermore, HLA-DR and DQ could not be associated with the time of onset of VLS, anatomical changes and localization of the skin lesions, and the response to topical glucocorticoids (34). In a cohort of Han Chinese women, the HLA-A*11, HLA-B*13, HLA-B*15, and HLA-DRB1*12 genotypes have been linked to a higher risk of VLS. Moreover, the women carrying HLA-A*11, HLA-B*15, HLA-B*35, and HLA-DRB1*12 genotypes were more susceptible to developing vulvar malignancy (35).

### 3.2. Epigenetic

Epigenetic changes in LS may potentially induce malignant transformation. At the beginning of the disease, LS lacks p53 and CDKN2A mutations, suggesting that the cell-cycle regulation is not appreciably altered (36). However, VLS is associated with altered isocitrate dehydrogenase, an enzyme responsible for DNA 5-hydroxymethylation patterns. As a consequence, the global methylation levels in the epidermis are diminished in VLS, and the UVA1 treatment could lead to a normalization of these levels (37). Nevertheless, the contribution of the epigenetics LS is enigmatic. It was described that a hypermethylation in p16INKa gene promoters could lead to an epigenetic silencing and, therefore, to an abnormal cell growth. This could be an early event in tandem with subsequent p53 somatic mutations, associated with tumor development and progression (38). Furthermore, the p16INKa hypermethylation was linked to vulvar carcinoma arising from LS, rather than to those not associated (39).

### 3.3. Immunology

In LS, there is an important T-cell infiltration limited to the dermis, composed mostly of CD8+ and Treg T-cells, and to a lesser extent, CD4+ T-cells (40). The involved cells express the chemokine receptors CXCR3 and CCR5 and lack CCR3 and CCR4, suggesting a Th1 profile. Th1 response intensifies through the production of interferon γ and the attraction of more Th1 cells. Other proinflammatory cytokines such as IL-1α, IL-7, IL-15, and TNF-α are upregulated in LS, whereas antiinflammatory cytokines (e.g., IL-10) are downregulated (39, 41). However, IL-4 levels are high in LS with congenital phimosis, a cytokine typically considered a marker of T helper 2 profile (42).

The small endogenous noncoding miR-155 plays a primordial role in regulating the homeostasis of the immune system and the sclerotic tissue formation. Activated immune cells in LS express miR-155 and, as a result, miR-155 enhances the Th1 differentiation. The high expression of miR-155 in CD4+ T cells reduces the Treg-cells-mediated suppression. As a consequence of miR-155 levels, the increased numbers of Treg cells in the dermis may not fully affect CD4+ T cells. Furthermore, the expression of Foxp3, a transcription factor of Treg cells, were significantly lower in the skin of VLS, compared to healthy vulvar skin. These results may lead to an impairment of the immune tolerance and, as a result, autoreactive CD4+ effector T cells may trigger an immune response against self-antigens (40, 41, 43).

The extracellular matrix protein 1 (EMC1) is a glycoprotein that binds different molecules of the basement membrane zone (BMZ) and dermis, being responsible for the structural organization and integrity in human skin. Autoantibodies against ECM1 and other antigens of the BMZ, i.e., BP180 and BP230, were found in gLS. The significance of the autoantibodies is unclear and, except for anti-EMC1 antibodies, they may only represent an epiphenomenon rather than a key-component of the LS pathogenesis (44, 45).

CD44 is a cell surface glycoprotein involved in cell adhesion and migration; it also acts as a hyaluronate receptor. The role of CD44 in keratinocytes is unclear. It was shown that the levels of CD44 are decreased in all layers of the epidermis in LS. The lack of CD44 may result in an abnormal dermal accumulation of hyaluronate in gLS and eLS (46, 47). On the other hand, other authors confirmed the high expression of pan CD44 around keratinocytes and in areas of inflammation in the dermis, but not in areas of sclerosis (48).

### 3.4. Fibroblast proliferation

Another characteristic of LS is the augmented collagen synthesis in the dermis, in particular, collagen I and III. The sclerotic tissue formation depends also on the increased expression of miR-155. As a consequence, the tumor suppressor genes FOXO3 and CDKN1B are downregulated, leading to fibroblast proliferation as well as persistence (49). Furthermore, in congenital phimosis due to LS, cytokines of the TGF-β superfamily are overexpressed. Among them, TGFβ-2 and BMP2 and their correspondent receptors play a central role in fibrosis control and tissue turnover (42).

ECM1 autoantibodies release the regulatory inhibition of metalloproteinase 9 (MMP9), thus enhancing the collagenase activity and, as a result, disrupting the BMZ (50). Nevertheless, the hyperactivity of MMP9 may cleave and activate TGF-β, hence the increased collagen synthesis (39). Apart from the increased levels of collagen I and III, an abnormal deposition of collagen V, a reduction of elastic fiber in the upper dermis as well as a low expression of ECM1 in the blood vessels of hyalinized tissue, were also observed (51).

The fibroblast activity may also be stimulated by galectin-7, a pro-apoptotic protein. In VLS, the epidermal levels of galectin-7 are increased, inhibiting keratinocyte viability and, thus, inducing epidermal atrophy (52). Galectin-7 could also act as paracrine in the fibroblast, enhancing the transcription of type I and III collagen and downregulating the cell growth rate (53).

### 3.5. Oxidative stress

Oxidative stress may be responsible for the pathogenesis, maintenance and progression of chronic inflammatory disease, including LS (54). Lipid peroxidation in keratinocytes, oxidative DNA damage and protein oxidation in areas of LS were associated with low concentrations of antioxidant enzymes, such as superoxide dismutase (55). The oxidative DNA damage in LS downregulates the expression of two cyclin-dependent kinase inhibitors p16I^INK4^ and p27^Kip1^. As a consequence, the inhibition of these two tumor suppressors releases the cell-cycle, thus contributing to the malignancy in LS (56).

Oxidative stress and inflammation may induce the overexpression of wild-type p53 in basal keratinocytes, independent from the LS subtype. Additionally, due to the development of sclerotic vessels and the poor oxygenation, the restriction of oxygen flow induces ischemic stress, intensifying the p53 overexpression (57). In contrast to vulvar carcinoma, chromosome 17p-linked loss of heterozygosity and p53 mutations are not characteristic in LS (58).

## 4. Risk factors

### 4.1. Trauma and chronic irritation

Chronic irritation and trauma play an important part in the development of LS. For instance, occlusion, scratching, friction and surgical procedures act as a Koebner phenomenon, resulting in the appearance of LS lesions (19). Urinary incontinence, multiparous status, scarce genital washing frequency and high BMI are associated with VLS in elderly women (59). In men, chronic irritative penile microincontinence, anatomical abnormalities (hypospadias), and interventions, e.g., cysto- and urethroscopies, radical prostatectomy, bladder surgery or penile prosthesis implantation are linked to MGLS (60, 61). LS affects uncircumcised men or boys with preputiolysis, but seldom circumcised males at birth (62).

There are also reports about koebnerization of eLS after insulin injections, influenza vaccination and intramuscular drug administration (63–65). Periostomal LS around urostomies are described as uncommon complications, possibly due to local trauma, occlusion and urine irritation (66). There are reports of LS after intimate body piercing (67, 68). Postirradiation eLS was described in patients treated for breast neoplasm, and VLS after vaginal cancer radiotherapy (69–71).

### 4.2. Hormones

The role of the hormones in LS is controversial. In the past, hypoestrogenism was considered a risk factor of VLS, due to its typical presentation during pre-puberty and postmenopause. However, this theory could not be proved (72). The expression of estrogen receptor (ER) isoforms in epidermis and dermis of VLS are comparable to those of healthy women. The isoform alpha of ER was absent in the fibromuscular layers, while the isoform beta was highly upregulated in VLS, as opposed to the absent expression of isoform beta in normal tissue. These variations in the ER isoforms may suggest that VLS is refractory to estrogen therapy (73).

Other studies speculated that a decreased 5 alpha-reductase activity could contribute to the pathogenesis of VLS by leading to low serum levels of dihydrotestosterone, free testosterone, and androstenedione (74). Furthermore, a low density of androgen receptors, both in gLS and eLS, could also induce a disease progression (75). Supporting this theory, it was observed that the use of oral contraceptives with anti-androgenic properties might trigger an early onset of VLS in susceptible young women (76). Nevertheless, the widespread topical treatment with testosterone became obsolete, as a consequence of the androgenic side effects and the therapy’s lack of effectiveness (9).

Contraception methods based on progesterone alone were, however, protective for VLS development, while this preventive benefit seemed to be lost when the contraceptive was combined with estrogens (12). Additionally, the topical treatment with progesterone 8% induced remission in 60% of premenopausal VLS but, in spite of that, ultrapotent TC are still superior in matters of therapeutic efficacy. (77).

### 4.3. Infections

There are no triggering infections associated with LS. However, there are many reports of the presence of HPV16 in the prepuce of not only adult men, but also in boys before having sexual intercourse (78, 79). In a case series of 329 patients, no correlation between MGLS and HPV infection was shown (61). Furthermore, in HPV16-associated MGLS cases, the role of the virus may be rather accidental than pathogenic, as also non-specific HPV-associated gene expression patterns have been found (80, 81).

*Borrelia burgdorferi* has also been postulated as a possible risk factor of LS and morphea, but this association remains obscure, as many studies showed no evidence for bacterial DNA in serological studies and skin biopsies specimens (82).

Many reports have suggested that hepatitis C virus (HCV) could also play a role in gLS and eLS (83, 84). In a lipidomic and metabolomic analysis, VLS correlated with an abnormal antivirus response due to the presence of HCV poly U/UC sequences (85). Nevertheless, the HCV serology screening in MGLS showed no seropositivity for the virus, thus assuming that it would unlikely play a pathogenic part in MGLS (86).

Circular RNA were shown to be closely related to the pathogenesis of different diseases and neoplasms. A recent study that aimed to investigate the differential expression profile of circular RNA in VLS observed an enrichment of the human T-cell leukemia virus (HTLV-1) signaling pathway, a retrovirus related to adult T-cell lymphoma/leukemia and chronic inflammatory diseases. The authors concluded that the HTLV-1 signaling pathway could be related to the occurrence and development of VLS (87).

### 4.4. Medications

The association of LS and medications is occasional. There are reports of blistering variants of LS, gLS and eLS with imatinib mesylate in patients treated for chronic myelogenous leukemia and gastrointestinal stromal tumor (88, 89). Carbamazepin has also been implicated in a case of generalized LS after 6 months duration of treatment (90).

There is a well-known association of autoimmune-induced diseases in cancer patients treated with immunotherapy and check-point inhibitors. In concrete, gLS was also reported in adult patients with malignancy, treated with pembrolizumab, nivolumab, or ipilimumab (91, 92). The reports indicated that the development of gLS usually occurred after 3 to 5 months of the beginning of therapy (91, 93).

There is an inverse relationship between VLS, ACE inhibitors and beta-blockers. While ACE inhibitors reduce the inflammatory cell infiltrate in the skin, beta-blockers reduce AMPc levels, induce keratinocyte proliferation and lymphocyte motility, which could impact on the clinical picture of VLS (94).

## 5. Clinics

LS lesions are flat, ivory-coloured, wax-textured spots, which may coalesce into crinkly thin or hyperkeratotic patches. Adjacent erythema and the Koebner phenomenon may also be observed. Other typical signs of LS are ecchymosis, excoriations and fissures. (3) LS can affect any part of skin or mucosa, however, in 85% of cases, the genital mucosa is involved, and extragenital lesions are seen only in around 15–20% of cases (23). Extragenital lesions often appear simultaneously with genital lesions, but in 6% of cases only extragenital forms have been reported (27).

### 5.1. Female lichen sclerosus

**Figure 2:**
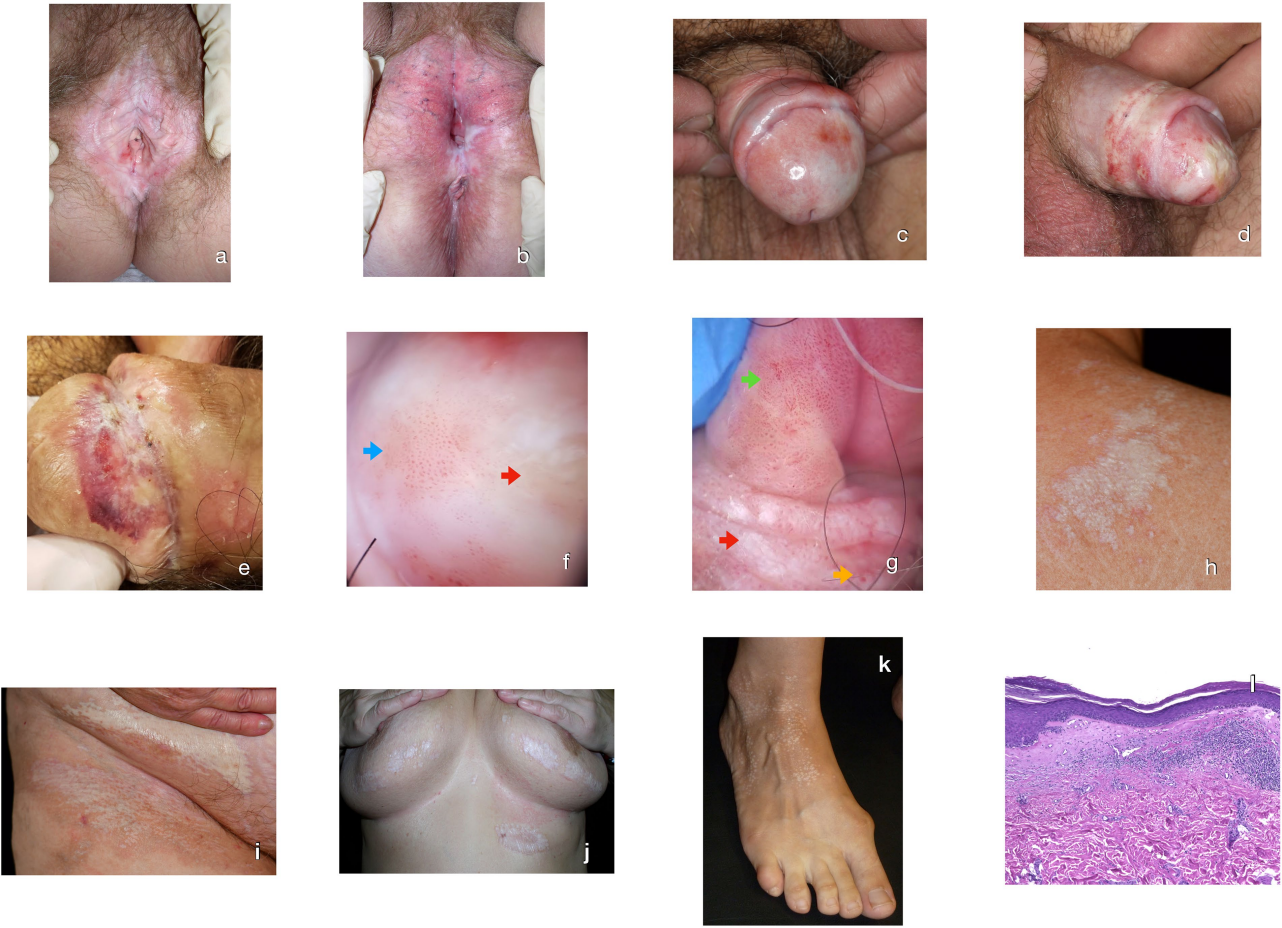
Clinical, dermoscopic and histological hallmarks of lichen sclerosus (LS). **(A)** Ivory-white wax-textured atrophic patches in the vulva. Agglutination, labial resorption, clitoral hood scarring. Fissures and erosions due to the narrowing of the vaginal introitus. **(B)** Erythema and anogenital scarring. Loss of the normal vulvar architecture with resorption of labia majora and minora. Ivory-white perineal spots perianal and scarring. **(C)** Hypopigmented macules, erythema and adhesions between glans and preputium. **(D)** Extensive scarring and atrophy of glans and partial loss of the balanopreputial sulcus. Ecchymotic and hypopigmented penile shaft. **(E)** Hemorrhagic variant of lichen sclerosus in glans penis over whitish scars, erosions and crusts. **(F)** Dermoscopy of LS in glans penis with structureless whitish and yellowish patches (red arrow), glomerular and dotted vessels (blue arrow). **(G)** Dermoscopy of LS in glans penis with penile intraepithelial neoplasia transformation. Glomerular vessels grouped over a pink and yellow surface, compatible with lichen sclerosus (green arrow). Structureless infiltrated whitish areas (red arrow) with glomerular and dotted vessels (yellow arrow), with histopathological confirmation of differentiated penile intraepithelial neoplasia. **(H)** Scapular ivory-white polygonal coalescing papules with adjacent erythema and comedo-like openings. **(I)** Whitish extensive macules, surrounded by hyperpigmentation, erythema and areas of ecchymosis on the groin. **(J)** Mammary and submammary ivory-white wax-textured atrophic coalescing spots with perilesional erythema and hyperpigmentation. **(K)** Small monomorph ivory-white wax-textured spots in extragenital lichen sclerosus. **(L)** The histopathology from a skin biopsy showing an atrophic epidermis with compact orthohyperkeratosis, hypergranulosis, degeneration of basal keratinocytes, and exocytosis. Under the thick basement membrane, there is a scarcely cellular zone of sclerosis. In the upper dermis, a superficial lichenoid inflammatory band and vasodilation with perivascular infiltrate are shown (H&E 40x).

Initial manifestations in the anogenital area in females can be nonspecific and include itch, burning sensation, as well as slight redness and swelling in the periclitoral area. Later on, affected skin becomes fragile and atrophic lesions, fissures and erosions may occur (Figure 2A). Fissures are often localized between clitoris and urethra and in the interlabial sulci, leading to dysuria (95). Due to intense pruritus, hyperkeratotic lesions and ecchymoses in the involved regions can be observed. Mostly, clitoral hood, labia minora, inner part of labia majora, perineum and perianal region is affected (Figure 2B), sometimes resembling “figure of eight,” also termed “keyhole” or “hourglass,” with involvement of vulvar and perianal regions. Progression of disease can lead to scarring, which is observed in 80% of adult female patients and 30% of girls (11). Scarring often results in fusion or even complete resorption of labia minora and loss of clitoral hood. In addition, narrowing of vaginal introitus can occasionally lead to dyspareunia, strongly affecting sexual life of the patients (5).

In prepubertal girls, the clinical symptoms are similar to that of adult females, which often present itch, soreness and sometimes dysuria. Typical clinical manifestations, such as fissures, erosions, vulvar and perianal bruising can be mistaken for sexual abuse (96). Perianal involvement and constipation, caused by painful anal fissures are frequently observed in girls. Importantly, the extent of the genital involvement does not directly correlate with the intensity of clinical symptoms, so not relevant lesions can cause significant complaints (72).

### 5.2. Male lichen sclerosus

Clinical manifestations of LS in boys and adult men are usually localized to glans penis and foreskin, whereas involvement of perianal area is rare. Typical symptoms include pruritus and soreness, sometimes accompanied by dysuria. Clinically, porcelain-like whitish sclerotic scarring on the distal portion of prepuce is usually observed (25). This scarring leads to phimosis in previously retractable foreskin, or adhesions of the foreskin to glans penis (Figure 2C) (3). In addition to preputial lesions, involvement of perifrenular region of glans is often seen, causing sclerotic frenulum breve (Figure 2D). In some cases, erosions, ulcers and even bullous lesions may be present. In around 17% of patients, external meatus urethrae can also be involved, which is often accompanied by dysuria and poor urinary stream. Sometimes meatal stenosis can appear years after the initial manifestation of LS, without any signs of persistent or recurrent LS (25, 97).

### 5.3. Extragenital lichen sclerosus

Extragenital manifestations are rare and found mostly in females, with female to male ratio ranging from 6:1 to 10:1 (27). Extent of the extragenital involvement vary from small well-defined area to widespread eruption, mostly localized to submammary area, neck, shoulders, inner thighs, wrists, upper back (Figures 2E–H) (98). Clinical lesions usually appear as asymptomatic or slightly pruritic ivory-white polygonal coalescing papules. Comedo-like plugs or evenly spaced dells, corresponding to appendageal ostia on the surface of the plaques are characteristic for extragenital LS. These dells and plugs may disappear with time, leaving smooth porcelain-white plaques (99). In some cases, the typical LS lesions are accompanied by telangiectasias, and hemorrhagic- or non-hemorrhagic bullae. Bullous lesions may lead to ulcerations or erosions that turn into white sclerotic plaques (100–102). The Koebner phenomenon is often observed, with extragenital manifestations of LS arising at pressure points, sites of surgical scars or traumas (19).

In rare cases, involvement of oral mucosa has been described, mostly affecting labial mucosa, followed by the buccal mucosa, and lip. Oral manifestations are asymptomatic well-demarcated macules with a whitish, ivory- or porcelain-white color. Pain, pruritus, and tightness when opening the mouth were described in a minority of the patients (103, 104).

## 6. Histology

Typical histological features of LS include orthohyperkeratosis, epidermal atrophy, degeneration of basal keratinocytes, homogenized collagen in upper dermis, and band-like and perivascular inflammatory infiltrate in the dermis (Figure 2I) (105, 106). However, the histological features in early stages are often difficult to distinguish among other lichenoid disorders (95). In early lesions, the initial dermoepidermal interface band of inflammation shifts gradually downwards into the dermis. As a result in later stages, the appendages disappear, due to the deposition of an altered extracellular matrix in the dermis. The destruction of the papillary capillaries starts early in LS, along with the thickening of the perivascular basement membrane. In the late stage, the capillaries are dilated and in resolved lesions, there is a complete absence of normal vessels (107). Some authors demonstrated an increased density of capillaries during the sclerotic stage of LS, in comparison with healthy controls (108). The BMZ demonstrates an alteration of the antigen expression, augmented collagen IV and VII, and loss of expression of hemidesmosomal and anchoring filament components. As a result of these changes, the BMZ is disrupted or some areas of BMZ are completely absent (109).

The immunohistochemistry approach shows a dense infiltrate of CD4+ and CD8+ T-cells, localized in the subepidermal inflammatory band. In certain cases, the T-cells also infiltrate the dermoepidermal junction, lower epidermis and dermis. CD68+ macrophages are present in the inflammatory band and also scattered throughout sclerotic regions. HLA-DR is also expressed, in variable percentages, in the inflammatory infiltrates and keratinocytes (48).

## 7. Associations

Autoimmune diseases are associated with LS in more than a quarter of the patients (110). In a large retrospective study, autoimmune thyroid diseases such as Hashimoto’s thyroiditis and Graves’ disease, antithyroid antibodies and elevated autoantibodies were more commonly associated with female LS (18.9%) than male patients (5.1%) (111). In a case–control study of 765 cases of VLS, it was found a 2.88-, 2.34-, and 2.05-fold increase in odds of having autoimmune thyroiditis, hypothyroidism, and hyperthyroidism, respectively. This study provides further evidence that screening for thyroid disorders in LS patients should be considered (112).

There are also reports from vitiligo, alopecia areata, rheumatoid arthritis, pernicious anemia, systemic lupus erythematosus, Sjögren syndrome, and multiple sclerosis (110, 111, 113–116). In 5.7% of patients, morphea coexists with LS, especially the circumscribed and generalized types (117). While in men morphea associates typically with eLS, in women the frequent relation with VLS obliges the inspection of the anogenital area (27).

Atopic dermatitis was found in 25% of boys who underwent circumcision due to gLS. The authors hypothesized that the disturbed atopic skin barrier could be more susceptible to LS triggering agents (118). In the case of psoriasis, the prevalence in the general population is 2%, while it rises to 7.5% in women suffering from VLS. Moreover, a total of 26.3% of the female psoriasis patients also had concomitantly LS (119).

Female patients with LS had an increased risk of developing metabolic syndrome in comparison to the healthy controls. They were also more likely to suffer from arterial hypertension, diabetes type 2, coronary artery and peripheral vascular diseases as well as to be prescribed statins. There were no differences in the body mass index, triglycerides or HDL levels, probably because of the statin prescription. The authors hypothesize that this relation could be due to an increased systemic inflammation and a decreased overall daily activity level (120).

## 8. Diagnosis

The diagnosis of LS in adults and children is typically clinical, involving a thorough medical history and physical examination. Photographic records are also recommendable to monitor therapy efficacy or disease progression (121). The clinical scoring system in VLS is a validated useful tool to ease the diagnosis and to evaluate related symptoms and the therapy response (Table 1) (122, 123). An autoimmune disease assessment should be conducted, especially in case of positive clinical features for thyroid autoimmune diseases, type 1 diabetes mellitus, rheumatoid arthritis or scleroderma (13, 124).

**Table 1:** Clinical score system in vulvar lichen sclerosus (122, 123).

Clinical signs	Grade 1 (moderate changes)	Grade 2 (severe changes)
Erosions	1–2 small erosions, almost not macroscopically visible	Macroscopically visible and/ or more than 2 or confluent lesions.
Hyperkeratosis	Affecting the vulva and perineum up to 10%	Affecting the vulva and perineum more than 10%
Fissures	Rhagades affecting the posterior introitus	Generalized vulvar rhagades
Agglutination*	Partially affecting preputium clitoridis and labia minora	Complete agglutination of both
Stenosis	Narrowing of the introitus which could still be passed by two fingers	A narrowing which could be passed by less than two fingers
Atrophy	Shrinkage of labia minora and clitoris	Labia minora and clítoris no longer visible

Grade 0 implicates normal anatomy. There is a 90% probability of VLS with a score > 4.

* Vulvar agglutination: partial or complete adherence of the labia minora or majora.

Histopathological assessment is mostly not necessary, as the clinical picture can be enough for the diagnosis. Nevertheless, there are many situations where an histological examination should always be considered (Table 2) (124). The biopsy should be performed from active sclerotic skin areas and erosions that do not improve after the therapy (13). Early, ulcerative or erythematous lesions are inadequate biopsy areas, as they present mostly an unspecified histological picture (105). Preceding treatment with topical corticosteroids can also erase typical histopathological changes (5).

**Table 2:** Recommendations for histological examination in lichen sclerosus (124).

1. Atypical clinical features
2. Presence of pigmented areas, in order to exclude abnormal melanocytic proliferation
3. Failure of first-line therapy to control symptoms and disease progression
4. Extragenital lichen sclerosus mimicking a morphea
5. Alternative or additional therapy to potent glucocorticoids
6. Young adult female patients with suspect of lichen sclerosus before beginning a treatment
7. Suspicion of a neoplastic change, such as areas of hyperkeratosis, erosion, erythema and papular lesions. A complete excision should be performed when the lesion is highly suspicious of squamous cell carcinoma
8. Confirmation of lichen sclerosus and exclusion of penile intraepithelial neoplasia after a circumcision.

Dermoscopy is a useful tool to support the non-invasive diagnosis of LS as well as to optimize the biopsy site (125). In VLS, the most prevalent feature is the presence of the structureless whitish or white-yellowish patches placed over a white atrophic background. These dermoscopic changes represent signs of dermal sclerosis and hyalinization. Another hallmark is the reduction of the vascular density compared to normal vulvar skin. The dermoscopy of VLS reveals irregular linear vessels in over 97% and dotted vessels in almost 45% of early stages VLS. In later stages, most vessels disappear as dermal fibrosis may exceed the vascular changes. Other dermoscopy signs include the scattered gray-blue dots, the comedo-like openings and the scales (125). In eLS, the most common dermoscopy hallmarks are the whitish structureless areas, comedo-like openings and telangiectasias and dotted vessels in the early stages. White chrysalis-like structures are not specific for eLS, but they have been documented in later stages (126). Rosettes or four-dot signs were also described in LS, however, they lack specificity (127). The dermoscopic clues of LS are further explained in Table 3 (125–130).

**Table 3:** Dermoscopy in lichen sclerosus (126–130).

Dermatoscopy sign	Description and presentation
Whitish structureless areas	Represents a sign of epidermal atrophy or hyperkeratosis, typically in early lesions. Together with comedo-like openings, the most statistically significant dermatoscopy signs.
Vascular structure	Irregular telangiectasias, comma shaped, hairpin-like, dotted vessels, as sign of dilated vessel in an atrophic epidermis, during early stages of the disease. Multicolored diffuse hemorrhagic area in bullous LS.
Comedo-like opening	Follicular plugins in the histopathologic study, present in early lesions. The ink test may be a useful technique to enhance the visualization.
Gray-blue dots	Melanophages in the upper dermis and at perifollicular sites
Rosettes	Four bright white dots or globules grouped like a four-leaf clover Unspecific, it can be observed in lichen planus, perniosis, apocrine hidrocystoma, and photocontact dermatitis, actinic keratosis and squamous cell carcinoma.
White chrysalis-like structures	Shiny, bright white, parallel, orthogonal or disordered linear streaks. Present in late lesions. Unspecific, also observed in morphea, lichen planus, dermatofibroma, basal cell carcinoma, Spitz nevus and melanoma

The reflectance confocal microscopy is another non-invasive technique for imaging skin *in vivo*. In MGLS, prominent fiber-like structures representing hyaline sclerosis can be observed in almost half of the patients, and an atypical honeycomb pattern could be related to penile intraepithelial neoplasia (131).

The impact of LS in the quality of life (QoL) as a whole, but also in relation to sexuality, should be assessed with validated tests. The dermatology life quality index (DLQI) measures the impact of the skin disease on different social areas and also covers symptomatology and response to the treatment. The female sexual functioning index (FSFI) is a self-reporting measure of sexual function in women with sexual arousal disorders and vulvodynia. The World Health Organization Five-Item Well-being Index is a short and generic global rating scale for subjective well being (132–135).

## 9. Differential diagnosis

### 9.1. Clinical differential diagnosis

A summary of differential diagnosis for gLS, eLS, and oral LS are listed in Table 4 (13). Anogenital lesions in women could be easily misdiagnosed for lichen planus, Candida vulvitis, postmenopausal atrophy and vitiligo, therefore, an extensive list of differential diagnoses should be considered. The most common form of lichen planus in the genital area is the erosive type, which may involve the vagina with symptomatic erythematous and friable lesions, inflammatory vaginal discharge, synechiae and obliteration. Candida vulvitis with or without vagina discharge can coexist with VLS and it might be treated repetitively before establishing the diagnosis of VLS. Even so, candida vulvitis can cause flares of VLS symptoms. Postmenopausal atrophy presents vaginal dryness, decreased lubrication, dyspareunia, discharge and urinary symptoms, with loss of vulvar tissue’ elasticity and fat. The clinical findings of vitiligo are asymptomatic demarcated chalk-white or milky macules that lack clinical signs of inflammation. Other differential diagnoses in VLS are lichen simplex chronicus, contact dermatitis, psoriasis, morphea, leukoplakia, extramammary Paget diseases, mucous membrane pemphigoid and vulvar intraepithelial neoplasia. In children, ecchymotic and bleeding could be a suspicion of sexual abuse (13, 136). Beside inflammatory and autoimmune diseases, in MGLS following diseases should be additionally considered as differential diagnosis: infectious and plasma cell balanitis, balanitis circinata, fixed drug eruption, penile neoplasms and in boys, physiologic phimosis (137, 138).

**Table 4:** Summary of clinical differential diagnoses in lichen sclerosus (LS) (13).

Vulvar LS	Male genital LS	Oral LS	Extragenital LS
Localized scleroderma/Morphea			
Lichen planus			
Vitiligo			
Autoimmune blistering diseases			
Contact dermatitis			
Intraepithelial neoplasia		Discoid lupus erythematosus	
Lichen simplex chronicus		Lichenoid reactions: GVHD	
Candidiasis			Hypopigmented MF
Leukoplakia			
Postmenopausal atrophy	Balanitis circumscripta plasmacellularis	White sponge nevus	

The differential diagnoses of eLS depend on the extent and morphology of the lesions. For the early indurated eLS lesions, discoid lupus erythematosus and morphea plaque type are clinically resemblant. While discoid lupus erythematosus presents scales, pigmentation and scarring on photoexposed skin, morphea has initially a violaceous halo. The late hypopigmented lesions should be differentiated from vitiligo, hypopigmented mycosis fungoides and atrophic lichen planus. The diagnosis of hypopigmented mycosis fungoides could be clinically challenging and it requires an histopathological confirmation. Beside vitiligo and morphea, generalized eLS may be confused for graft-*vs*-host disease. Bullous LS may pose a diagnostic challenge in bullous lichen planus, autoimmune blistering diseases, bullous scleroderma, bullous lupus erythematosus and bullous insect bite reactions (27, 139).

Oral LS could be mistaken for many other mucous lesions. Oral lichen planus presents white reticular plaques, usually with a bilateral and symmetrical distribution. Lichenoid reactions show a reticular, atrophic or ulcerative lesions, often asymmetrical, with a history of an implicated medication, dental restorative material, or graft-versus-host disease. Leukoplakia are white plaques that cannot be categorized as any other disease, and premalignant lesions usually emerge as a proliferative verrucous leukoplakia. Candida infection presents with white patches or pseudomembranes that can be removed, revealing underneath an erythematous mucosa. Other differential diagnoses include vitiligo, localized scleroderma, fibrous scar, submucous fibrosis, white sponge nevus and discoid lupus erythematosus (7).

### 9.2. Dermoscopic differential diagnosis

Morphea is one of the most challenging differential diagnoses of LS by dermoscopy. While in LS white-yellowish patches are the most common dermoscopy feature, morphea presents white fibrotic beams. Both structures are similar, however, the white fibrotic beams tend to be smaller, more opaque with less defined margins, compared to the white-yellowish patches in LS. Vascular components such as linear-irregular or dotted vessels are to be found in both pathologies. LS often displays follicular plugging, which may correlate with the rosettes. Morphea lacks comedo-like openings, scaling and hemorrhagic areas. The presence of structureless or reticular brownish areas is more common in morphea, while pigmented dots may be found in both. The rosette sign, along with whitish plaque and comedo-like openings, may lead to an earlier diagnosis of LS. Dermoscopy differential diagnoses in gLS and eLS are listed in Table 5 (140–145).

**Table 5:** Summary of dermoscopy differential diagnoses of genital and extragenital lichen sclerosus (140–145).

Morphea	Presence of white fibrotic beams, structureless or reticular brownish areas. Absence of comedo-like openings, scaling and hemorrhagic areas
Vitiligo	Reduced or absence of pigment network, intralesional spots of residual pigmentation and telangiectasias
Lichen planus	Wickham striae (inconstant and peripheral in vulvar lichen planus), thick linear, irregular, hairpin and spermatozoa-like vessels, dull and intense red background and blue dots or globules
Psoriasis	Light red background, uniform-distributed dotted and tortuous vessels. Diffuse lamellar white scales are typical in extragenital psoriasis.
Lichen simplex chronicus	White-grayish background with diffuse arranged linear, serpentine, and dotted vascularization
Plasma cell mucositis	Serpentine, convoluted, and chalice-shaped vessels over red-yellowish to orange-brownish structureless areas
Granuloma annulare (patch-type)	White and yellowish-orange structureless areas, blurry vessels with variable morphology
Mycosis fungoides	Orange-yellow patches, geometric perifollicular white scales and white patches as well as short, fine and linear vessels
Necrobiosis lipoidica	Comma-shaped (early lesions) or elongated, branching, and focused serpentine (advanced lesions) vessels, yellowish-orange or whitish-pinkish background

## 10. Management

Every case of gLS should be treated, in order to preserve QoL and to prevent scarring, anatomical, sexual and urinary dysfunction (9, 124). The therapeutic strategy should be discussed with the patient, as there are many options that have been explored to achieve clinical remission and prevent progression. A list of non-pharmacological recommendations as well as the proposed schema with TC in LS are enumerated in Table 6 (9, 124). A list of the ongoing clinical trials is tabulated in Table 7.

**Table 6:** Summary of recommendations for adults with lichen sclerosus (LS; Modified from the British Association of Dermatologists guidelines, 2018) (124).

Recommendations for all people with lichen sclerosus	
GPP	Management of LS by healthcare professional experienced in treating the condition
GPP	Full clinical history including urinary symptoms, dyspareunia and psychosexual issues. Documentation of anatomical changes at baseline
GPP	Avoid irritants and fragranced products, use an emollient soap substitute/cream, dry after passing urine, use a moisturizer or yellow soft paraffin, stop smoking to reduce the risk of cancer, use of lubricant to avoid painful intercourse, lifelong regular self-examination.
GPP	Discuss the amount of topical treatment, the site of application and the safe use of topical steroids in lichen sclerosus.
⬆	Consider intralesional triamcinolone in LS with topical steroid-resistant or hyperkeratotic areas, when malignancy has been excluded by biopsy.
GPP	All people treated for LS should be followed up
**Recommendations for female adult patients**	
⬆⬆	Offer clobetasol propionate 0,05% ointment for 3 months in anogenital LS (once a day the first month, every other day the second month and twice weekly for the last month)
⬆⬆	Offer continued use of clobetasol propionate 0,05% for ongoing active disease
⬆	Consider an individualized treatment regimen of topical steroid to maintain disease control and prevent scarring in female patients with ongoing active LS disease despite good compliance.
GPP	Consider referral to a vulval specialist in all female patients, when the patient is not responding to topical steroid, or if surgical management is being considered.
**Recommendations for male adult patients**	
⬆⬆	Offer all male patients with genital LS clobetasol propionate 0,05% ointment once daily for 1–3 months
GPP	Consider a repeat course of topical treatment for 1–3 months in those who relapse.
⬆⬆	Offer all male patients with phimosis not responding to ultrapotent topical steroid after 1–3 months referral to an experienced urologist for circumcision.
GPP	Offer clobetasol propionate 0,05% ointment applied once daily *via* cotton wool swab for meatal involvement by LS with meatal dilator for 1–3 months prior to referral to a urologist.
GPP	Offer male patients with urinary symptoms, urethral stricture due to LS or who have failed to topical steroids and/or circumcision referral for a urology opinion and further investigation and management of lower urinary tract symptoms and treatment with other surgical options.
GPP	Advise obese male patients with LS and a buried penis to lose weight

Level of recommendation: ⬆⬆: Strong recommendation for the use of an intervention. ⬆: Weak recommendation for the use of an intervention. GPP: Good practice point recommendations are derived from informal consensus.

**Table 7:** Selected interventional clinical trials in female anogenital lichen sclerosus.

Study	Intervention	Population	Study design	Phase	Status
NCT05010421	Topical clobetasol 0.05% vs. non-ablative CO_2_ Laser	>18 y.o. *n* = 198	Randomized, parallel assignment, open label	3	Recruiting
NCT03665584	Fractionated CO_2_ laser vs. sham laser	>18 y.o. *n* = 40	Randomized, triple masking	n/a	Completed
NCT03961126	Injection of autologous fatty tissue + autologous platelet-rich plasma vs. topical clobetasol 0.05%	18–70 y.o. *n* = 20	Randomized, parallel assignment, open label	2	Completed
NCT05228483	Photodynamic treatment monotherapy or combined with CO_2_ fractional laser vs. topical mometasone furoate 0.1%	18–60 y.o. *n* = 134	Randomized, parallel assignment, single masking	n/a	Recruiting
NCT03525522	Nd:YAG laser (1,064 nm) treatment vs. topical betamethasone	>18 y.o. *n* = 40	Randomized, parallel assignment, single masking	n/a	Unknown
NCT05243563	Fractionated CO_2_ laser with or without topical clobetasol 0.05%	18–85 y.o. *n* = 52	Randomized, parallel assignment, open label	2/3	Recruiting
NCT03045172	Platelet rich plasma injections vs. placebo	>18 y.o. *n* = 29	Randomized, parallel assignment, single masking	n/a	Completed
NCT02416531	Topical clobetasol 0.05% vs. photodynamic therapy or low level laser therapy	>18 y.o. *n* = 30	Randomized, parallel assignment, open label	n/a	Completed
NCT04134494	Ablative Fractional 2,940 nm Laser	>18 y.o. *n* = 14	Single group assignment, open label	n/a	Active, not recruiting
NCT04967170	Autologous Platelet-Rich Plasma vs. sham procedure	>18 y.o. *n* = 60	Randomized, parallel assignment, single masking	1/2	Recruiting
NCT05364515	Topical clobetasol	>18 y.o. *n* = 30	Randomized, parallel assignment, open label	3	Not yet recruiting
NCT04107454	Microablative fractional CO_2_ laser vs. sham laser	>18 y.o. *n* = 64	Randomized, parallel assignment, single masking	n/a	Active, not recruiting
NCT03926299	Dual laser treatment with thermal Nd:YAG and ablative Er:YAG vs. topical clobetasol propionate 0.05%	>18 y.o. *n* = 66	Randomized, parallel assignment, open label	n/a	Active, not recruiting
NCT05250466	Radiofrequency technology vs. electrical stimulation	18–60 y.o.	Randomized, parallel assignment, open label	n/a	Not yet recruiting
NCT05593445	Ruxolitinib 1.5% cream twice a day for 12 weeks vs. vehicle cream followed by ruxolitinb 1.5% cream BID twice a day for 12 weeks for both groups.	>18 y.o. *n* = 60	Randomized, parallel assignment, triple masking	2	Recruiting

A search on clinicaltrials.gov was conducted on September 21st, 2022 and updated on January 13th, 2023 with the key word “lichen sclerosus.” A total of 39 clinical studies were identified. Abbreviations: n/a: not applicable; y.o.: years old.

### 10.1. Topical treatment

A summary of topical and intralesional therapies in LS with recommendation grade and evidence level is described in Table 8.

**Table 8:** Summary of topical and intralesional therapies in lichen sclerosus (3, 9, 77).

Drugs	Dosage & observations	Evidence level	Grade of recommendation
**First-line therapy**			
Very potent and potent topical corticosteroids	Gold standard; clobetasol propionate 0.05% or mometasone furoate 0.1% ointment. Dosage: Once a day for 1 to 3 months in men or once a day for the first month, on alternate days for the second month and twice weekly for the third month in women. Maintenance therapy: Once or twice weekly	1 to 2	A
Intralesional glucocorticoids	Positive effect on resistant pruritus and signs of disease progression in case of intolerance to topical steroid or treatment failure. Dosage: Monthly intra/subdermal triamcinolone acetonide or dexamethasone solution for 3–4 months. Maintenance therapy required	1+	B
**Second-line therapy**			
Topical tacrolimus 0.1%	Effective and safe, but not superior to TC Dosage: once or twice daily for 4–8 weeks	2+	C
Topical tacrolimus 0.03%	Effective and safe in children Dosage: once or twice daily for 4–8 weeks	3	D
Pimecrolimus 1% cream (adults)	Effective and safe, but not superior to TC Dosage: once or twice daily for 4–8 weeks	1+	B
Pimecrolimus 1% cream (children)	Alternative to TC in girls Dosage: once or twice daily for 4–8 weeks	3	D
**Third-line therapy**			
Topical retinoids	Beneficial effect, but lacks proper studies. Useful on hyperkeratotic lesions. Dosage: on alternate days for 12–24 weeks.	3	D
**Miscellaneous therapies**			
Vitamin E & Moisturizers	Symptom relief after an initial treatment with TC. Vitamin E has not a beneficial effect over emollients.	2+ to 3	D
Topical estrogens	Not recommended	-	-
Topical testosterone	Not recommended, not superior to TC	1+	A
Topical progesterone 2 and 8%	Not recommended, not superior to TC	1+	A
Topical ciclosporin	Effective in a minority of patients	3	D
Intralesional adalimumab	Last resource treatment	3	D

#### 10.1.1. Glucocorticoids

Topical glucocorticosteroids are the first-line therapy in gLS (9). Most studies recommend clobetasol propionate 0.05% ointment (CP) daily, as it improves symptoms and signs in three quarters of VLS (146). A dose of 0.5 grams daily during 1 to 3 months applied to the affected area should be sufficient. The risk of adverse effects is low and some authors described no predisposition to infections, contact dermatitis and worsening of the skin atrophy (147). In a randomized trial in VLS, mometasone furoate 0.1% ointment (MF) showed a comparable efficacy and tolerability to CP (148). In a retrospective study of MGLS, topical CP during 3 months in decreasing application frequency was successful in almost 60% of MGLS, avoiding the subsequent necessity of circumcision (61).

In girls, CP 0.05% applied 3 months is also the most effective treatment to induce remission and manage the symptoms. Despite the good response, recurrences were reported in two-thirds of the girls within 1 year, requiring a new cycle of CP for 3 months and/or intermittent maintenance (149). In boys, the use of TC such as MF or betamethasone cream prevents circumcision in up to 35% of the cases (150, 151). The preputioplasty combined with intralesional corticosteroid is an effective alternative in boys, however, with a higher rate of relapse compared to circumcision (152).

Long-term treatment with ultrapotent or potent TC appears to be safe and effective and improves the QoL. Maintenance treatment has shown a greater effectiveness and it is usually more often required in VLS than in MGLS (9). A proactive maintenance treatment should be recommended after achieving remission, in order to prevent recurrence and malignity (3). In a cohort of 327 VLS patients, it was observed a symptom relief in 96% treated with ultrapotent TC, 66% became symptom free, while only 23% had completely reversed skin signs (11).

#### 10.1.2. Calcineurin inhibitors

Calcineurin inhibitors, namely tacrolimus 0.1% ointment or pimecrolimus 1% cream once or twice a day for 1–2 months, can be considered as an off-label alternative in case of failure or intolerance to CP (3). Both in adults and children, topical calcineurin inhibitors are effective and safe alternatives to TC, nevertheless, CP seems to be more effective and should remain as the first-line therapy (153, 154). A relationship between calcineurin inhibitors and malignancy in LS has not yet been established (9).

### 10.2. UV light treatment

#### 10.2.1. Phototherapy

UVA1 phototherapy is a potential first-line therapy for eLS, with an evidence level 1+ and recommendation grade B (9, 155). There are also reports of VLS treated successfully with narrowband UVB, UVA1 and topical PUVA phototherapy (156–158). However, phototherapy for VLS should be only indicated in case of failure to standard therapies, as it is not superior to TC regarding symptom relief, QoL and practicability (159). Furthermore, the well-known development of skin malignancy after phototherapy could be inconvenient in genital areas, as the risk of cancer *per se* in LS is increased (9).

#### 10.2.2. Photodynamic therapy

Photodynamic therapy (PDT) with topical 5-aminolevulinic acid is a valid option to treat incoercible pruritus in VLS, when other therapies have failed (9). However, clinical and histological improvements are controversial (160). Objectively, some cases reported healing of superficial erosions and improvement of the clinical signs, whereas others demonstrated no clinical changes after PDT (161–164). Moreover, no improvement was revealed in the histopathological findings, while other authors described an augmented apoptosis and resolution of the chronic inflammation (165–167). Regarding adverse effects, burning sensation during PDT and lubrication disorders following the therapy were reported (165, 168). Recurrence of the VLS was observed by many authors after 3 to 9 months of therapeutic response (164, 169).

### 10.3. Systemic treatment

Systemic therapy is sporadically indicated and only recommended in widespread eLS or LS refractory to the standard topical treatment. In a retrospective study, pulsed high-dose corticosteroids combined with low-dose methotrexate therapy improved the clinical condition in refractory generalized eLS (170). Systemic retinoids such as etretinate and acitretin were effective in eLS, severe gLS or when other therapies failed to control the disease (171, 172). A list of systemic treatments with the recommended doses are enumerated in Table 9 (170–181).

**Table 9:** Summary of effective systemic therapies in recalcitrant lichen sclerosus (170–181).

Drugs	Dosis	Efficacy	Side effects	Grade of recommendation	Level of evidence
Pulsed steroid and methotrexate	Methylprednisolone 1,000 mg/d IV for three days monthly + methotrexate 15 mg/w PO > 6 months	Decrease of the clinical score after 3 months of treatment in 85,7% of patients	Methylprednisolone: hypertension, arrhythmia, hyperglycemia, hypokalemia, abnormal behavior, risk of infections.	3	D
Retinoids	Acitretin 20–30 mg/d PO 16 weeks Etretinate 1 mg/kg/d PO 14–18 weeks	Acitretin efficacy group 64 vs. 25% placebo group Etretinate: Improvement of symptoms in 75% of patients.	Cheilitis and dry skin (+++), palmoplantar peeling, hepatic alterations, hypertriglyceridemia, abdominal pain, dizziness, hair loss, teratogenesis.	1+	B
Ciclosporin	3–4 mg/kg/d PO for 3 months	Decrease of the clinical score after 1 month and symptoms after 3 months in five patients.	Nausea, hypertrichosis, mucositis	3	D
Methotrexate	10–15 mg/w PO 6 months	Clinical improvement in 75% of patients after a median of 3 months	MethotrexateNausea, headache, increase of liver enzyme levels; Gastrointestinal discomfort, fatigue, hair loss.	3	D
Hydroxycarbamide	1 g/d PO 1 month	Improvement of symptoms in a single case report.	Gastrointestinal discomfort neutropenia, Carcinogenicity	3	D
Hydroxychloroquine	200 mg/d PO 3 months	Case report with resolution of the symptoms and modest clinical improvement	Nausea, diarrhea, skin rash, retinopathy, hemolytic anemia,	3	D
Antibiotics	Penicillin G benzathine 2.4 M units IM or Ceftriaxone 1 g IM every 2 weeks, next once a month	A 53,3% disease clearing with or without residual atrophy, after 3 to 9 months.	Injection site pain, type I hypersensitivity reactions, *C. difficile*-associated diarrhea	3	D
Sulphasalazine	1–2 g/d PO (long-term therapy)	Case report. Reduction of the skin infiltration after 1 month	Headache, nausea, fever, skin rash, and reversible infertility in men, pancreatitis.	3	D
Vitamin D	Calcitriol 0.5 μg/d PO 6 months	Case report with clinical and histological improvement after 4–6 months	Hypercalciuria	3	D
Baricitinib	2 mg/d PO combined with PUVA twice-weekly	Case report in eLS with improvement of pigmentation and atrophy after three months	Not described in this case report.	3	D

Grade recommendations and levels of evidence are explained in Supplementary Table 1.

### 10.4. Surgical treatment

There is no surgical first-line therapy for adult MGLS and recommendations are based on expert opinion or non-analytical studies (9, 182). Therefore, the initial treatment should include an ultrapotent TC for mild cases, and if the patient remains symptomatic after a sufficient conservative treatment, they should be offered a surgical option (9). Some of the proposed surgical techniques are circumcision, dilating or surgically correcting meatal stenosis, urethroplasty techniques and glans resurfacing with CO_2_ laser (Table 10) (183). An early complete circumcision in boys improves the prognosis of MGLS restricted to foreskin and glans, and it could also resolve the disease completely without any additional postoperative antiinflammatory treatment (184, 185). Recurrences are seldom after a complete foreskin removal, but the residual foreskin in partial circumcisions showed a recurrent disease in 50% of the patients (25). Glans and meatus involvement are negative prognostic factors for meatal stenosis and proximal urethral strictures (9, 186). Periodic dilations of strictures are a surgical option unlikely to be a permanent solution for MGLS. An indication for meatotomy is the meatal stenosis with uroflowmetry with a plateau phase under 10 ml/min (25, 187). Strictures longer than 2 cm, recurrent or penile urethral strictures could benefit from primary urethroplasty (188).

**Table 10:** Summary of surgical treatments in genital lichen sclerosus (9, 183).

Surgical procedure	Indication	Observations	Evidence level	Recommendation grade
**Male genital lichen sclerosus**				
Circumcision	Persistent phimosis	Risk of recurrence in obese boys or hypospadias repair	3	D
Meatal Dilatation, meatotomy & meatoplasty	Meatal stenosis	Appears with LS or after months of the circumcision	3	D
Urethroplasty & urethral reconstruction with mucosal graft	Urethral stenosis	Pedicle penile skin urethroplasty: high risk of relapse. Replacement of diseased urethra and mucosal graft: no recurrences.	3	D
**Female genital lichen sclerosus**				
Vulvectomy	Vulvar malignancy and severe dysfunctional anatomy	Mutilating procedure with recurrence rate over 50%	4	D
Urethroplasty	Urethral stenosis	Reconstruction with oral mucosal graft	3	D
Clitoral circumcision	Clitoral phimosis	CO_2_-Laser division or hydrodissection & reverse V-plasty	3	D
Perineotomy & reconstruction	Introitus stenosis	Relief dyspareunia Perioperative anti-inflammatory treatment and dilators to prevent restenosis	3	D
De-adhesiolysis, dissection or Z-plasty	Labial adhesions		3	D

Surgery in VLS patients should be indicated in selected cases (Table 10) (9). Labial agglutination and clitoral hood scarring refractory to conservative treatments should be surgically corrected, in order to avoid obstructive urinary symptoms and for cosmetic reasons (189). Female sexual dysfunction due to severe clitoral phimosis can be treated with clitoral circumcision (190). Median perineotomy and perineoplasty are reserved for severe cases of introital stenosis, thus achieving an improvement in QoL (191, 192). Vulvectomy in LS is an invasive procedure with poor outcome and recurrence rate of up to 40–50% (193, 194). Hence, vulvectomy should only be performed in case of vulvar malignancy (9).

### 10.5. Miscellaneous treatments

#### 10.5.1. Laser therapy

Laser therapy is an emerging therapeutic in gLS, despite the poor evidence and the lack of long-term data to support it (9, 195). Carbon-dioxide laser ablation may improve symptoms, signs and QoL in gLS and eLS. Long term remission of up to 14 years was described after CO_2_ therapy in a single arm trial, but only symptomatic patients were invited to a clinical examination to confirm the presence of LS (196). Nevertheless, some authors conclude that CO_2_ laser is not only superior to high-potent TC, but also not effective enough as monotherapy for VLS and it should be performed only as adjuvant therapy, in addition to TC (195, 197).

#### 10.5.2. Focused ultrasound

High focused ultrasound (HIFU) has been demonstrated to be an effective treatment for VLS in pediatric and adolescent patients. HIFU is a nonsurgical treatment that relieves pruritus and stimulates cell proliferation and revascularization in order to repair damaged tissue (198). The common adverse effects are blistering and ulcers due to possible skin burns. After a follow-up of 5 years, the total response rate was 75%, and the recurrence rate was 12.5% (199). HIFU could be considered as an alternative if the standard treatment fails (9).

#### 10.5.3. Regenerative therapies

The regenerative therapies could be considered as a therapeutic option for complications such as atrophy and scarring, when the first-line therapy is unresponsive. For instance, platelet rich plasma (PRP) seeks to repair damaged tissue and restore the skin function. PRP promotes angiogenesis, cell proliferation and differentiation and regulates the inflammatory cascades in LS (200). PRP improves QoL and the objective parameters in gLS (201, 202). However, the reports on PRP have a poor level of evidence due to a lack of standardization for PRP preparation and application (203).

## 11. Complications

### 11.1. Scarring

Lichen sclerosus in women leads to skin fragility, bleeding, chronic fissuring scars and secondary infections. It may evolve to a fusion of the labias, complete loss of the vulvar architecture, and narrowing of the vaginal introitus, preventing normal sexual intercourse. Furthermore, chronic anterior scarring can trap the clitoris within the clitoral hood, leading to phimosis and painful clitoral engorgement during arousal (clitoral pseudocyst) (13, 204, 205). After a long-term severe scarring may occur urinary retention, anal stenosis, obstruction and constipation (13).

In MGLS, preputial scarring leads to a frenulum contracture and a progressive fibrous phimosis with trans- and subcoronal adhesions. Frenulum scarring alters significantly QoL in men and a frenuloplasty in the context of complete circumcision should be recommended (124). Urethral involvement and urinary microincontinence is also reported in MGLS (9, 183, 206). To prevent further scarring after surgery, ultrapotent TC should be regularly applied, particularly around the coronal sulcus, if LS is still active (124).

### 11.2. Malignancy

LS in genital areas has a risk of neoplastic transformation to squamous cell carcinoma (SCC) (207, 208). Vulvar SCC was observed in 3.5 to 7% of women with VLS, while up to 65% of vulvar carcinomas arise on a background of VLS (209). Hence, early detection of premalignant lesions and a lifelong follow-up are required (13, 210). In a retrospective study, the neoplasia incidence rate in 976 women with VLS was 8.1 per 1,000 person-years, and the cumulative probability of progression to vulvar cancer escalates from 1.2% at 2 years to 36.8% at 25 years (211). In another cohort, VLS predisposed women under 70 years of age for vulvar SCC, with a attributable risk of 98% (209). Penile SCC was estimated in 4 to 13.4% of MGLS cases (210, 212, 213). Twelve percent of all penile SCC are entirely due to MGLS, whilst it was found that 29.4% of them arose in combination with histologically confirmed HPV (214).

The pathomechanism of malignancy in LS is unknown, but it is presumed that chronic inflammation, oxidative stress and single base substitution mutations at C742T and G818C in p53 could play an important role (215). Most cases of LS associated with SCC are not related to HPV infection and when detected, it may be a non-oncogenic type (216). Moreover, HPV-negative SCC associated with MGLS expresses p53 and lacks p16^ink4a^ overexpression (217). While some authors mentioned that ultrapotent TC could act as risk for cancer due to local immunosuppression, one study shows a statistically significant lesser likelihood to develop malignancies when TC were regularly used as a prophylactic measure in asymptomatic VLS (9, 209, 218, 219).

There are also sporadic reports of concomitant melanoma, basal cell and Merkel carcinoma with LS, without any increased frequency (13). Other gynecologic malignancies have been reported in VLS. In a retrospective survey, the association between LS and endometrial and ovarian carcinoma was statistically significant, whereas colon cancer was not related to VLS. Furthermore, all patients with ovarian cancer had a history of LS. The association with breast cancer is controversial, as one work group found a higher frequency in LS, while another could not replicate this link (220, 221). When gynecologic cancers precedes VLS, the increased level of circulating extracellular matrix protein 1, estrogen status and radiotherapy could play a part in the development of VLS (220).

### 11.3. Psychosexual impact

LS negatively impacts on the psychosexual health of both women and men (9, 61). The late diagnosis of LS leads to frustration and delays in starting an adequate treatment (222). Both sexes experience a reduction in QoL in LS, but they might experience the symptomatology differently (223). Through DLQI assessment, VLS diminishes QoL and correlates with sexual functioning impairment, mostly attributed to the physical symptoms of LS and the feelings of embarrassment, anxiety, and stigma around open discussion of genital conditions (222, 223). More than a half of male and female LS patients experienced pain during sexual intercourse, but complaints about LS were higher in women (223, 224). The QoL in men after circumcision improved remarkably; however and despite adequate treatments, psychosexual issues may still persist in women, particularly in those with a higher degree of disease severity (225, 226).

### 11.4. Sensory abnormality

Vulvodynia, burning sensation around the glans and urethral meatus may occur after inflammatory diseases. The symptoms remain usually long after the disease has remitted. Neuropathic pain does not improve with TC and other approaches should be considered (124).

## 12. Follow-up

The follow-up visits in LS enables the assessment of the response to treatment and gives the opportunity to educate the patient as well as to rule out complications. The efficacy of the therapy should be evaluated by the improvement of the symptoms and clinical manifestations with photographic documentation, performed with every visit. For both uncomplicated VLS and MGLS, the first follow-up visit after 3 months, assesses the response and the proper use of CT, while a second follow-up visit 6 months later, gives the chance to discuss remaining problems. Patients with active ongoing disease require a long-term follow-up. Even after many years of resolved gLS, patients should seek referral to specialists in case of signs of recurrent disease (3, 124).

Men with persistent disease and phimosis, who do not respond to high potent TC after 3 months of treatment, should be reviewed for surgery. Histopathological studies should be performed in case of suspicious lesions. Patients with urinary and sexual symptoms should be referred to a urology specialist to measure flow rate and postvoid residual volume in order to identify and treat urethral stricture or meatal stenosis (227).

A long-term follow-up by secondary-care specialists should be proposed in VLS, when the patients had an atypical clinical course, uncontrolled symptoms with TC or previous cancer. Persistent erythematous lesions, erosions, ulcerations and hyperkeratotic plaques should be biopsied. Female patients who underwent surgery due to anatomical changes need a postoperative follow-up and TC in order to prevent recurrences (228).

## 13. Outlook

LS is an under-recognized and misdiagnosed dermatosis with a not-fully understood pathogenesis. The late recognition of LS may alter the QoL, lead to disfiguring anatomical changes and increase the risk of genital cancer. Although laser-based, regenerative therapies and more innovative approaches with topical JAK inhibitors are promising, ultrapotent topical corticosteroids are still the most commonly used first-line treatment. Periodic controls are necessary for the early detection of characteristic complications. Hopefully, with the emergence of new treatment options and proper high-quality studies, the pathogenetic and therapeutic landscape of LS will improve in the near future.

## Author contributions

All authors listed have made a substantial, direct, and intellectual contribution to the work and approved it for publication.

## Funding

Funding sources from Cluster of Excellence Precision Medicine in Chronic Inflammation (EXC 2167) and the Research Training Group Autoimmune Pre-Disease (GRK 2633), all from the Deutsche Forschungsgemeinschaft; and the Schleswig-Holstein Excellence-Chair Program from the State of Schleswig Holstein.

## Conflict of interest

DT: Honoraria for participation on advisory boards, as a speaker and for consultancy from AbbVie, Almirall, Boehringer Ingelheim, Bristol Myers Squibb, Eli Lilly, Galapagos, Janssen, LEO Pharma, Morphosis, Novartis, Pfizer, Regeneron, Samsung, Sandoz, Sanofi Genzyme, and UCB Pharma; research grants received from LEO Pharma, and Novartis.

The remaining authors declare that the research was conducted in the absence of any commercial or financial relationships that could be construed as a potential conflict of interest.

## Publisher’s note

All claims expressed in this article are solely those of the authors and do not necessarily represent those of their affiliated organizations, or those of the publisher, the editors and the reviewers. Any product that may be evaluated in this article, or claim that may be made by its manufacturer, is not guaranteed or endorsed by the publisher.
