# Supplementary material for: Lichen sclerosus: The 2023 update
Source: Front Med (Lausanne). 2023 Feb 16;10:1106318. doi: 10.3389/fmed.2023.1106318 (PMC9978401; doi:10.3389/fmed.2023.1106318)
Supplement: Supplementary file 1 [file Table_1.pdf]

Supplementary Table 1 - Level of evidence and grade of recommendation (Evaluation of articles according to GRADE: www.gradeworkinggroup.org)

**Level of evidence**	**Type of evidence**
1++	High-quality meta-analyses, systematic reviews of RCTs, ***or*** RCTs with a very low risk of bias
1+	Well-conducted meta-analyses, systematic reviews of RCTs, ***or*** RCTs with a low risk of bias
1-	Meta-analyses, systematic reviews of RCTs, ***or*** RCTs with a high risk of bias *
2++	High-quality systematic reviews of case–control or cohort studies ***or*** high-quality case–control or cohort studies with a very low risk of confounding, bias or chance and a high probability that the relationship is causal
2+	Well-conducted case–control or cohort studies with a low risk of confounding, bias or chance and a moderate probability that the relationship is causal
2-	Case–control or cohort studies with a high risk of confounding, bias or chance and a significant risk that the relationship is not causal *
3	Non-analytical studies (for example, case reports, case series)
4	Expert opinion, formal consensus
**Grade of recommendation**	**Evidence**
A	At least one meta-analysis, systematic review, or RCT rated as 1++, and directly applicable to the target population, ***or*** a systematic review of RCTs or a body of evidence consisting principally of studies rated as 1+, directly applicable to the target population and demonstrating overall consistency of results
B	A body of evidence including studies rated as 2++, directly applicable to the target population and demonstrating overall consistency of results, ***or*** extrapolated evidence from studies rated as 1++ or 1+
C	A body of evidence including studies rated as 2+ , directly applicable to the target population and demonstrating overall consistency of results, ***or*** extrapolated evidence from studies rated as 2++

D	Evidence level 3 or 4, ***or*** extrapolated evidence from studies rated as 2+ , ***or*** formal consensus
D (GPP)	A good practice point (GPP) is a recommendation for best practice based on the experience of the guideline development group

* Studies with a level of evidence '–' should not be used as a basis for making a recommendation RCT, randomized controlled trial.
